# Culturable bacterial diversity from the chestnut (*Castanea sativa* Mill.) phyllosphere and antagonism against the fungi causing the chestnut blight and ink diseases

**DOI:** 10.3934/microbiol.2017.2.293

**Published:** 2017-05-03

**Authors:** Angel Valverde, María González-Tirante, Marisol Medina-Sierra, Raúl Rivas, Ignacio Santa-Regina, José M. Igual

**Affiliations:** 1 Instituto de Recursos Naturales y Agrobiología de Salamanca (IRNASA), Consejo Superior de Investigaciones Científicas (CSIC), 37008 Salamanca, Spain; 2 Departamento de Microbiología y Genética, Universidad de Salamanca, 37007 Salamanca, Spain; 3 Unidad Asociada Universidad de Salamanca-CSIC (IRNASA), Salamanca, Spain; 4 Department of Genetics, Centre for Microbial Ecology and Genomics, University of Pretoria, Pretoria 0028, South Africa; 5 Estación Biológica de Doñana, 41001 Sevilla, Spain; 6 Escuela de Producción Agropecuaria, Group GRICA (Grupo de Investigación en Ciencias Agrarias), Facultad de Ciencias Agrarias, Universidad de Antioquia, Medellín, Colombia

**Keywords:** *Castanea sativa*, *Cryphonectria parasitica*, *Phytophthora cinnamomi*, phyllosphere, bacterial diversity, bacterial-fungal antagonism

## Abstract

The phyllosphere supports a large and complex bacterial community that varies both across plant species and geographical locations. Phyllosphere bacteria can have important effects on plant health. The sweet chestnut (*Castanea sativa* Mill.) is an economically important tree species affected worldwide by the fungal pathogens *Cryphonectria parasitica* and *Phytophthora cinnamomi*. We examined the culturable phyllosphere bacterial community of the sweet chestnut at two nearby locations in Central Spain in order to know its geographical variability and to explore its potential as source of biological control agents against these two pathogenic fungi. The bacterial diversity at strain level was high but it varied significantly between locations; however, phylotype richness and diversity were more comparable. The isolates were affiliated with the phyla *Actinobacteria*, *Firmicutes* and *Proteobacteria*. Most of them were members of recognized bacterial species, with a notable proportion of representative of the genera *Dietzia* and *Lonsdalea*, but a small fraction of the strains revealed the existence of several potential novel species or even genera. Antagonism tests showed the occurrence in the chestnut phyllosphere of bacterial strains potentially useful as biological control agents against the two pathogenic fungi, some of which belong to species never before described as fungal antagonists. Chestnut phyllosphere, therefore, contains a great diversity of culturable bacteria and may represent an untapped source of potential biocontrol agents against the fungi causing blight and ink diseases of this tree species.

## 1. Introduction

The sweet chestnut (*Castanea sativa* Mill.) has been systematically cultivated since the Middle Age throughout southern Europe [1], from the Caspian Sea to the Atlantic Ocean. It covers more than 1.7 million ha in a discontinuous, scattered range, occupying coppices and orchards on acid soils [2]. Chestnut is an economically important species used for both nut and wood production, but its by-products has also a good profile of bioactive compounds with antioxidant, anticarcinogenic and cardioprotective properties [3], as well as it is a good contributor to carbon sequestration and a valuable source of biomass. Furthermore, the chestnut landscape offers great benefits in terms of social welfare [4].

The principal sanitary threats to chestnut trees in Europe are of fungal origin. *Cryphonectria parasitica* is the ascomycete responsible for chestnut blight, causing cankers that kill branches and trunks. The oomycete *Phytophthora cinnamomi* causes the ink disease of chestnut that is characterized by obstruction of the xylem vessels and exudation of blackish sap (due to air oxidation of tannins), resulting in a progressive decline of the uppermost shoots and finally of the whole crown [5]. Chestnut blight is currently present in Central and South Europe, East and West Coasts of North America, East Asia, and Tunisia [6]. Phytophthoras have been found to be widespread in Southern Europe and England [7],[8]. Drought and heat stresses seem to exacerbate the progress of both diseases [9],[10]. Moreover, the predicted global climate warming would be favourable for these two pathogenic fungal species [11], thus worsening the perspectives for chestnut forestry worldwide.

The aerial leaf surface or phyllosphere is colonized by variety of different organisms, including many different genera of bacteria, filamentous fungi, yeasts, algae, and, less frequently, protozoa and nematodes. Bacteria are by far the most abundant inhabitants of the phyllosphere, often being found in numbers averaging 10^6^ to 10^7^ cells/cm^2^ (up to 10^8^ cells/cm^2^) of leaf [12],[13]. Historically, many of the studies on phyllosphere microbial communities have focused on bacteria known to be plant pathogens [14],[15]. Much less is understood about the identity or properties of the numerous non-pathogenic microbes that inhabit the phyllosphere. It is known that many phyllosphere bacteria produce phytohormones that have the potential to affect plant development and productivity [16], fix nitrogen [17],[18] or can degrade organic pollutants [19]. Also, such colonists apparently play important roles in modulating population sizes of deleterious organisms [20],[21],[22], and some are potential source of pharmacological agents [23] or are being exploited as biological control agents (BCAs) for plant diseases [24]. In this sense, there is optimism concerning future prospects of application of bacteria for biological control of foliar plant pathogens and, in fact, some bacterial strains are already marketed in products for use in many crops, including tree species [25].

Although the phyllosphere of a few representative species of Mediterranean forest have been investigated as natural habitats for epiphytic microorganisms in general, and bacteria in particular [17],[26],[27], there is very scarce information regarding the phyllosphere of sweet chestnut [28]. In this study, we therefore aimed at identifying the culturable bacterial community of the chestnut phyllosphere and at exploring its potential as source of biological control agents against *C. parasitica* and *P. cinnamomi*, the most important fungal pathogens of such an economically essential tree.

## 2. Materials and Methods

### 2.1. Site description, leaf sampling and isolation of phyllosphere bacteria

Leaf samples were taken from chestnut trees growing at two locations in the mountain range of “Sierra de Tamames-Las Quilamas”, Salamanca province, Spain. These two sampling sites, here termed C and CH, occupy opposite slopes of a hill, at an altitude of approximately 1100 m above sea level. The site C (40° 35.13′ N; 5° 56.41′ W) is a chestnut orchard for fruit located on the north side of the hill. The site CH (40° 34.35′ N; 5° 57.06′ W) corresponds to a forest stand for wood production, which is predominantly oriented towards the southeast. At the time of sampling, chestnut trees at both locations were free of fungal diseases. The soil in this area is an Umbric Regosol with a pH of 5.35–5.26 and an organic matter content of 5.8–4.5%. This region has a humid Mediterranean climate with mean annual temperature of around 10 °C and mean annual precipitation of around 1300 mm [29].

At each stand, 100 individual leaves were taken randomly from 10 different chestnut trees (10 leaves per tree) and pooled together to form a sample. Each pooled leaf sample was placed in a sterile plastic bag and transported to the laboratory on ice for immediate processing. The leaves samples were weighed and placed in 1000 ml of sterile prechilled buffer [0.1 M potassium phosphate (pH 7.0), 0.1% peptone], following which bacterial cells were removed by 7 min of sonication in an ultrasonic bath. Samples (0.1 ml) from appropriate dilutions of the sonicate were plated on Tryptic Soy Agar (TSA) amended with 0.15 mg of cycloheximide per ml to inhibit fungal growth. Bacterial colonies were counted following 72 to 96 h of incubation at 28 °C. Numbers of viable bacteria were determined by counting the number of colonies on appropriate plates, with three replicate plates per dilution.

Selection of bacterial isolates was performed randomly following the methodology described in Jacobs and Sundin [30]. Briefly, the plates were placed on a numbered grid (0 to 50), three numbers were randomly chosen and two or three colonies in the chosen grids were selected from the dilution plates used to make the bacterial counts. Isolates were subcultured through two rounds of single-colony purification and subsequently stored at –80 °C in 25% glycerol.

### 2.2. Total DNA fingerprinting and 16S rRNA gene partial sequence analyses

Isolated colonies were subjected to two primers random amplified polymorphic DNA (TP-RAPD) analysis and accordingly to TP-RAPD profiles “phylotypes” were defined. TP-RAPD patterns were analyzed according to the method described by Rivas et al. [31] using the primers 879F (5′-GCCTGGGGAGTACGGCCGCA-3′) and 1522R (5′-AAGGAGGTGATCCANCCRCA-3′). The diversity of strains within each TP-RAPD group was then assessed by random amplified polymorphic DNA (RAPD) fingerprinting using the primer M13 (5′-GAGGGTGGCGGTTCT-3′) as described previously [32].

One representative strain from each TP-RAPD group was selected for taxonomic identification through 16S rDNA sequencing. Amplification of 16S rRNA genes was performed as specified by Rivas et al. [31]. The first 600–800 bp of these amplified 16S rDNA fragments were sequenced. The identification of phylogenetic neighbors was initially carried out by the BLAST [33] and FASTA [34] programs. The 50 sequences with the highest scores were then selected for the calculation of pairwise sequence similarity using global alignment algorithm, which was implemented at the EzBioCloud server [35]. For phylogenetic analysis, sequences were aligned using the Clustal X software [36]. The phylogenetic tree was inferred using the neighbour-joining method [37]. The distances were calculated according to Kimura's two-parameter method [38]. Bootstrap analysis was based on 1000 resamplings. The MEGA4 package [39] was used for all analyses.

### 2.3. Inhibition of fungal growth by bacteria

All bacterial isolates were screened for their ability to inhibit the growth of the phytopathogenic fungi *Cryphonectria parasitica* and *Phytophthora cinnamomi*. The strain of *C. parasitica* used in this study was SA1, kindly provided by Prof. Díez-Casero, ETSIIA, University of Valladolid, Spain. This strain belongs to the vegetative compatibility group EU11 and was isolated from infected chestnut trees in the vicinity of the sampling site of this study. The strain of *P. cinnamomi* was obtained from the Spanish Type Culture Collection (http://www.cect.org) under accession number CECT 2965 (=IMI 077375). This strain was isolated from *Castanea sativa* in France.

The antifungal activity of the bacterial isolates was assayed on potato dextrose agar (PDA) plates according to the method described by Ansari and co-workers [40]. In brief, a loopful of a log phase bacterial culture was spread on a line 3-cm away from the edge of the PDA Petri plates (9-cm diameter). The plates were inoculated with one mycelial plug (0.5-cm diameter) of *C. parasitica* or *P. cinnamomi* taken with the help of a sterilized cork borer from the margins of 7-day old cultures grown on PDA. The plug was placed 3-cm away from the bacterial strike and 3-cm from the wall of the dish. Control plates were inoculated with mycelial plugs (3 cm from the Petri dish wall) only. Each test had three replications. The dishes were incubated in the dark at 26 °C for five to seven days until the fungal growth reached the edge of the plate. Antifungal activity was monitored at regular intervals of time by measuring fungal growth radii along three axes: one axis perpendicular to the bacterial streak and one another axis at both left and right sides forming 45° with the first one (45°, 90° and 135° from the center of the fungal plug towards the bacterial streak).

### 2.4. IAA production by bacteria

Bacterial cultures were tested on TSA plates amended with 5 mM L-tryptophan as described by Bric et al. [41]. Briefly, agar plates (9-cm diameter) were inoculated with toothpicks into a grid pattern from TSA cultures. Each inoculated plate was overlaid with an 82-mm diameter nitrocellulose membrane. Grid plates were overlaid immediately after inoculation and incubated at 28 °C until colonies reached 0.5 to 2 mm in diameter. After an appropriate incubation period, the membrane was removed from the plate and treated with Salkowski reagent. Membranes were saturated by overlaying on a reagent-saturated Whatman no. 2 filter paper. The reaction was allowed to proceed until adequate color development. Bacteria producing IAA were identified by the formation of a characteristic red halo within the membrane immediately surrounding the colony. Colonies were assayed in triplicate and the experiment was repeated two times.

### 2.5. Statistical analysis

Data from antifungal activity assays were statistically analyzed by repeated measures two-way ANOVA and Bonferroni-adjusted post-hoc test for pairwise comparisons, using SPSS software (v21.0). The estimated species richness was determined through ACE and Chao1 non-parametric estimators, using SPADE [42]. SPADE was also used for the determination of Shannon's and the reciprocal of Simpson's indices (maximum likelihood estimators) and estimated sample coverage. Rarefaction curves were computed using the EstimateS program [43].

### 2.6. Nucleotide sequence accession numbers

One sequence for each phylotype (31 in total) reported in this study has been deposited in GenBank under accession numbers EU099594, GQ183833–GQ183862.

## 3. Results

### 3.1. Culturable bacterial community structure on the phyllosphere of chestnut trees

The number of total culturable bacteria ranged 1.7–3.2 × 10^6^ CFU g^−1^ fresh weight. After isolation a total of 2.1% of the isolates failed to grow during the subculturing process or could not be cultured following storage at –80 °C; these isolates were removed from further analysis. Thus, a final total of 227 isolates were studied, 117 from the sampling site C and 110 from the CH site.

TP-RAPD fingerprinting was used as shortcut technique for grouping the phyllosphere isolates in a taxonomically meaningful way. The diversity of strains within each TP-RAPD group was then assessed by RAPD fingerprinting using a primer derived from the M13 bacteriophage (M13-RAPD). Therefore, these two genetic fingerprinting techniques were used to define OTUs (operational taxonomic units) at two levels of phylogenetic resolution, sub-phylotype or strain level (M13-RAPD patterns) and phylotype level (TP-RAPD patterns).

**Figure 1.**
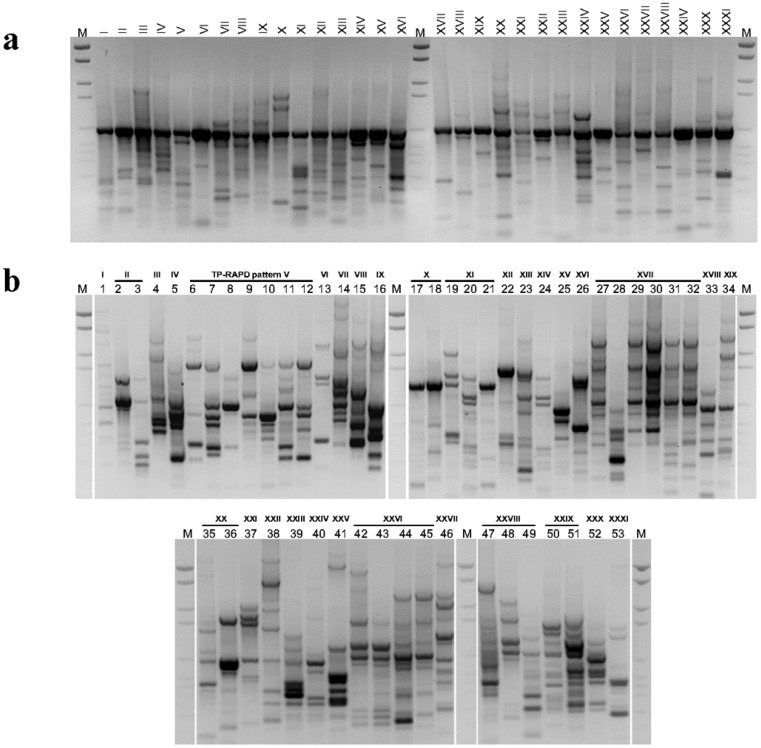
TP-RAPD (a) and M13-RAPD (b) profiles separated on ethidium bromide stained agarose gel (inverse display) of representative bacterial strains isolated from the chestnut phyllosphere. TP-RAPD patterns are noted in roman numerals. Representative strains and their taxonomic designations are given in Table 2. The DNA molecular weight marker (lane M) is Standard VI (Boehringer-Roche, Indianapolis, IN, USA) with 2176, 1766, 1230, 1033, 653, 517, 453, 394, 298, 234, and 154 bp.

**Figure 2.**
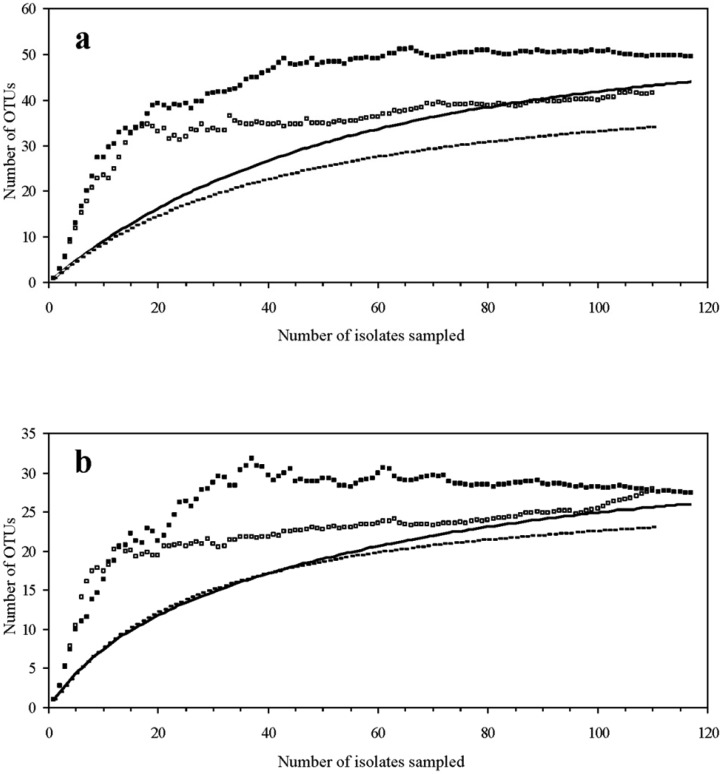
Observed and estimated OTU richness of culturable bacterial populations from the chestnut phyllosphere at the C and CH locations versus sampling size. OTUs as defined by M13-RAPD (a) and TP-RAPD (b) fingerprinting. The Chao1 estimated OTU richness (C ▪; CH □) and rarefaction curves (C —— ; CH – – – –) averaged over 50 simulations are shown.

Among the 227 bacterial isolates, there were observed 31 TP-RAPD patterns (Figure 1a) and 53 M13-RAPD patterns (Figure 1b), indicating the presence of genetically different strains (different M13-RAPD patterns) within the same bacterial phylotype (same TP-RAPD pattern). For instance, among the isolates showing the TP-RAPD patterns V, XVII and XXVI there were observed 7, 6 and 4 different M13-RAPD patterns, respectively (Figure 1b). Broken down by sampling site, 44 and 34 different bacterial strains were distinguished among the isolates from sites C and CH, respectively. These strains distributed into 26 (site C) and 23 (site CH) phylotypes. After 117 (site C) and 110 (site CH) sampling events, rarefaction curves did not reach an asymptote either for strains or phylotypes (Figure 2a, 2b), but they were far beyond the linear range. Moreover, Good's coverage values (Table 1) were high at both the strain (M13-RAPD patterns) and phylotype (TP-RAPD patterns) resolution levels. The estimated OTU richness in the samples was determined through Chao1 and ACE non-parametric estimators. Chao1 estimates a mean phylotype number (TP-RAPD patterns) of 27–28 and 28–36, and a mean strain number (M13-RAPD patterns) of 50–51 and 42–44 for the bacterial assemblages C and CH, respectively. ACE estimators were roughly comparable to Chao1 estimators (Table 1).

**Table 1.** Comparison of the OTUs richness from the culturable bacterial community in the phyllosphere of *Castanea sativa* Mill. at the two studied locations.

				Estimated OTUs ^b^			
DNA fingerprinting	Location	No. of OTUs	Good's coverage ^a^	Chao1	Chao1 b-c ^c^	ACE	ACE-1 ^d^
TP-RAPD	C	26	0.949	28 ± 2 (26–37)	27 ± 2 (26–35)	31 ± 4 (27–44)	32 ± 5 (27–49)
	CH	23	0.955	36 ± 17 (25–117)	28 ± 6 (24–55)	26 ± 3 (24–37)	26 ± 3 (24–40)
M13-RAPD	C	44	0.889	51 ± 5 (46–68)	50 ± 4 (46–65)	51 ± 4 (46–66)	52 ± 5 (46–69)
	CH	34	0.909	44 ± 8 (36–76)	42 ± 6 (36–66)	41 ± 5 (36–57)	43 ± 6 (37–65)

^a^ Good's Coverage = [1 – (*n*/N)]; where *n* is the number of OTUs represented by one bacterial strain and N is the total number of bacterial strains.

^b^ Estimators of species richness. Mean values (±SE) are shown, with lower and upper 95% confidence intervals given in parentheses.

^c^ A bias-corrected form for the Chao1.

^d^ A modified ACE for highly-heterogeneous communities.

**Figure 3.**
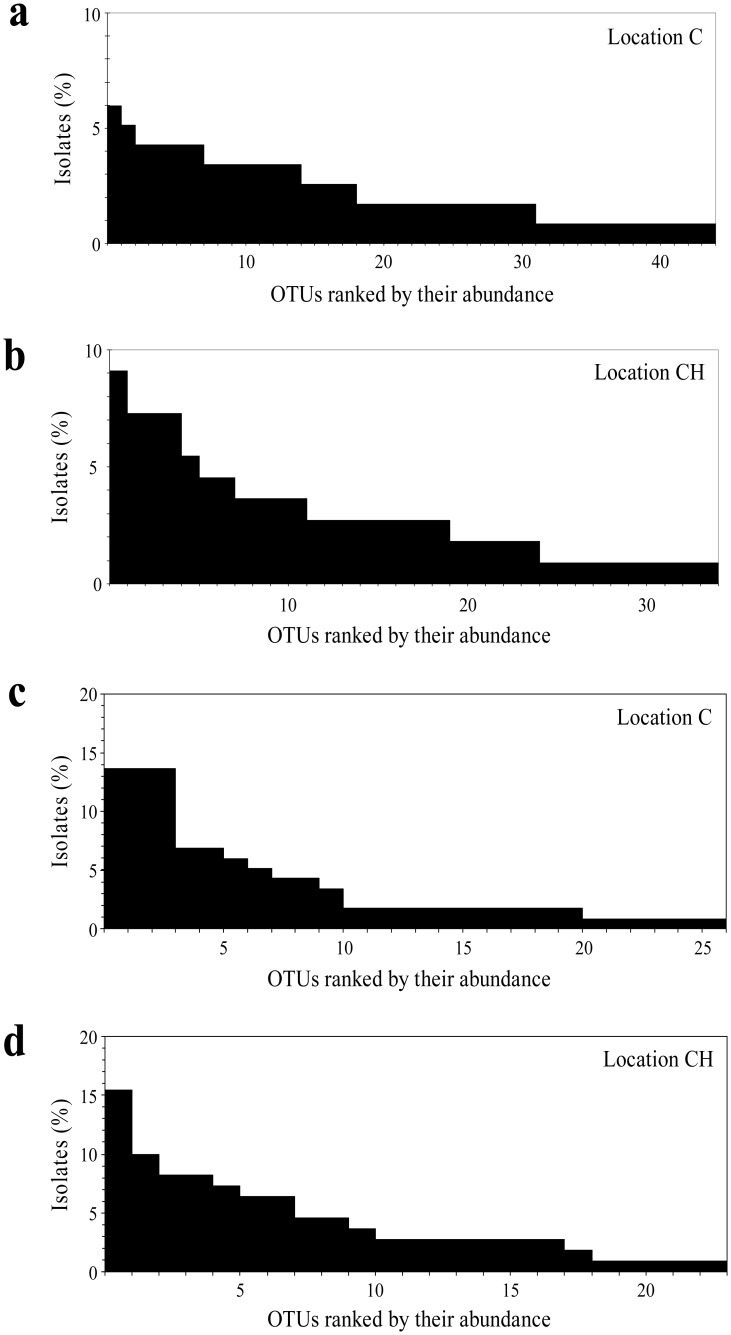
Rank-order plot showing the relative frequency of each OTU as defined by M13-RAPD (a, b) and TP-RAPD (c, d) patterns in the phyllosphere of chestnut trees growing at locations C (a, c) and CH (b, d).

From all the strains and phylotypes detected in this study, only 47% of the strains (25 out of 53) and 58% of the phylotypes (18 out of 31) were found at both sampling sites. Both in the bacterial assemblage C and in the bacterial assemblage CH, isolate richness was not equally distributed either among genotypes (M13-RAPD patterns) or phylotypes (TP-RAPD patterns). Thus, approximately 50% of the isolates were contained in only about a third of the genotypes (27–35%) (Figure 3a, 3b) and a fifth of the phylotypes (15–22%) (Figure 3c, 3d), while around another third of both the genotypes (30–38%) and the phylotypes (38–39%) accounted for only 10% of all isolates.

**Table 2.** Estimated diversity indices for the culturable bacterial community in the phyllosphere of *Castanea sativa* Mill. at the two studied locations. Mean values (±SE) are shown, with lower and upper 95% confidence intervals given in parentheses.

	Shannon ^a^			1/D ^b^		

DNA fingerprinting	Location	MLE	MLE b-c	Jackknife	MVUE	MLE
TP-RAPD	C	2.83 ± 0.08 (2.67–2.99)	2.96 ± 0.11 (2.75–3.16)	2.97 ± 0.10 (2.78–3.17)	13.91 ± 0.13 (13.65–14.16)	12.52 ± 0.13 (12.27–12.77)
	CH	2.85 ± 0.07 (2.71–2.98)	2.96 ± 0.10 (2.77–3.15)	2.98 ± 0.08 (2.81–3.14)	15.73 ± 0.15 (15.45–16.02)	13.88 ± 0.13 (13.62–14.14)
M13-RAPD	C	3.61 ± 0.05 (3.51–3.71)	3.83 ± 0.06 (3.70–3.95)	3.87 ± 0.08 (3.72–4.02)	44.35 ± 0.08 (44.19–44.51)	32.36 ± 0.07 (32.22–32.50)
	CH	3.29 ± 0.06 (3.17–3.41)	3.47 ± 0.08 (3.31–3.64)	3.50 ± 0.09 (3.33–3.67)	27.63 ± 0.11 (27.41–27.85)	22.24 ± 0.10 (22.05–22.44)

^a^ MLE: maximum likelihood estimator; MLE bc: bias-corrected maximum likelihood estimator.

^b^ Reciprocal of Simpson's index. MVUE: minimum variance unbiased estimator; MLE: maximum likelihood estimator.

**Figure 4.**
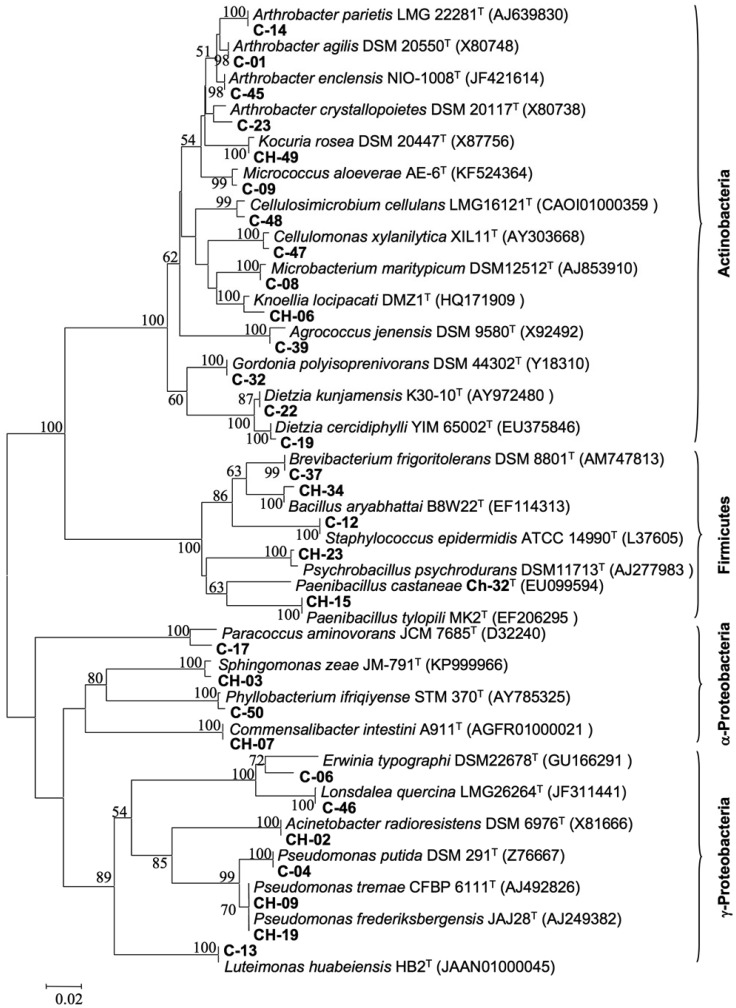
Phylogenetic neighbor-joining tree based on partial 16S rRNA gene sequences showing the relationship between representative strains of each TP-RAPD pattern (in bold types) and closely related type strains. GenBank accession numbers are in parentheses. The significance of each branch is indicated by a bootstrap value calculated for 1000 subsets; only boostrap values greater than 50% are shown. Bar, 2 substitutions per 100 nt.

Statistical estimates of bacterial diversity were obtained for each bacterial assemblage using the Shannon and Simpson diversity indices (Table 2). Both indices indicated that the diversity at genotype level (M13-RAPD patterns) was significantly greater in the C assemblage than in the CH assemblage.

### 3.2. Taxonomic diversity of phyllospheric bacteria

One strain representative of each distinct TP-RAPD pattern was identified after partial 16S rRNA gene sequencing using the EzBioCloud server (http://www.ezbiocloud.net) [35]. Analysis of the sequences indicated that the majority of isolates matched members of recognized bacterial species (16S rDNA sequence similarities of ≥99%) (Table 3). However, a small fraction of the strains (Table 3) has 16S rRNA gene sequence similarities lower than 98.7% to any of the currently recognized bacterial species. As that is the threshold value to differentiate bacterial species [44], it likely indicates the existence in the chestnut phyllosphere of several potential novel species, although a polyphasic characterization and full-length sequencing of 16S rRNA genes still have to be carried out to fully clarify their taxonomic status [45].

The majority of the isolates (58%) were affiliated with the two Gram-positive phyla, the Actinobacteria with 110 strains (48%) and the Firmicutes with 21 strains (10%). From the phylum Actinobacteria there were representative strains of the genera *Dietzia* (34 strains), *Micrococcus* (33 strains), *Arthrobacter* (13 strains), *Kocuria* (9 strains), *Knoellia* (4 strains), *Cellulomonas* (4 strains), *Brevibacterium* (3 strains), *Cellulosimicrobium* (3 strains), *Gordonia* (3 strains), *Microbacterium* (3 strains) and *Agrococcus* (1 strain). From the phylum Firmicutes, there were isolates belonging to the genera *Paenibacillus* (11 strains), *Bacillus* (2 strains), *Psychrobacillus* (5 strains) and *Staphylococcus* (3 strains). After the Actinobacteria, γ-Proteobacteria were the next most abundant group (36%), with representatives of the genera *Pseudomonas* (44 strains), *Lonsdalea* (25 strains), *Luteimonas* (7 cepas) and *Erwinia* (4 strains) and *Acinetobacter* (2 strains). Finally, the α-Proteobacteria were found to be the less abundant bacterial group in the chestnut phyllosphere (6%), which included isolates belonging to the genera *Phyllobacterium* (5 strains), *Sphingomonas* (4 strains), *Paracoccus* (3 strains) and *Commensalibacter* (2 strains). If we should focus on site of sampling in terms of incidence, four genera were well represented at both sampling locations, namely *Micrococcus*, *Dietzia*, *Lonsdalea* and *Pseudomonas*; while isolates representative of genera such as *Brevibacterium*, *Agrococcus*, *Paracoccus*, *Commensalibacter* and *Staphylococcus* were much less numerous and only found in one of the two locations.

### 3.3. Antifungal activities of the phyllosphere bacteria

All the bacterial strains isolated in this work from chestnut phyllosphere were prescreened for antagonism against the fungal pathogens causing chestnut blight (*C. parasitica*) and ink (*P. cinnamomi*) diseases by dual-plate assay on PDA plates. Most of the bacterial isolates showed little to no inhibition of fungal growth, sometimes fungal mycelia even grew over the bacterial colonies, which suggest that these strains did not produce any diffusible amensalistic substance that could influence the fungal growth. Only four and six out of the 227 bacterial strains showed substantial antifungal activity against *C. parasitica* and *P. cinnamomi*, respectively. In such cases, antagonism on agar was probably limited to amensalism, because competition for nutrients rarely, if ever, occurs on media with high nutrient levels like PDA. These strains were selected for further detailed analysis.

**Table 3.** Taxonomic identification of representative strains of each TP-RAPD pattern. Identification based on the 16S rRNA gene sequence using EzBioCloud server (http://www.ezbiocloud.net).

					RAPD group		

Strain	Closest related type strain	Accession no.	Identity (%)	Taxonomic group	TP-	M13-	No. strains
C-39	*Agrococcus jenensis* DSM 9580^T^	X92492	99.5	Actinobacteria	15	25	1
C-01	*Arthrobacter agilis* KCTC 3200^T^	X80748	99.8	Actinobacteria	1	1	2
C-23	*Arthrobacter crystallopoietes* DSM 20117^T^	X80738	98.8	Actinobacteria	12	22	6
C-45	*Arthrobacter* *enclensis* NIO-1008^T^	JF421614	99.7	Actinobacteria	16	26	3
C-14	*Arthrobacter* *parietis* LMG 22281^T^	AJ639830	100.0	Actinobacteria	8	15	2
C-37	*Brevibacterium* *frigoritolerans* DSM8801^T^	AM747813	100.0	Firmicutes	14	24	3
C-47	*Cellulomonas* *xylanilytica* XIL11^T^	AY303668	99.4	Actinobacteria	18	33	4
C-48	*Cellulosimicrobium* *cellulans* LMG16121^T^	CAOI01000359	99.5	Actinobacteria	19	34	3
C-19	*Dietzia* *cercidiphylli* YIM65002^T^	EU375846	99.7	Actinobacteria	10	17,18	15
C-22	*Dietzia* *kunjamensis* K30-10^T^	AY972480	100.0	Actinobacteria	11	19,20,21	19
C-32	*Gordonia* *polyisoprenivorans* NBRC16320^T^	Y18310	99.2	Actinobacteria	13	23	3
CH-06	*Knoellia* *locipacati* DMZ1^T^	HQ171909	98.9	Actinobacteria	24	40	4
CH-49	*Kocuria* *rosea* DSM20447^T^	X87756	99.5	Actinobacteria	20	35.36	9
C-08	*Microbacterium* *maritypicum* DSM12512^T^	AJ853910	99.7	Actinobacteria	4	5	3
C-09	*Micrococcus* *aloeverae* AE-6^T^	KF524364	99.5	Actinobacteria	5	6,7,8,9,10,11,12	33
CH-07	*Commensalibacter* *intestini* A911^T^	AGFR01000021	100.0	α-Proteobacteria	25	41	2
C-17	*Paracoccus* *aminovorans* DSM8537^T^	D32240	98.2	α-Proteobacteria	9	16	3
C-50	*Phyllobacterium* *ifriqiyense* STM370^T^	AY785325	99.7	α-Proteobacteria	21	37	5
CH-03	*Sphingomonas* *zeae* JM-791^T^	KP999966	100.0	α-Proteobacteria	23	39	4
CH-34	*Bacillus* *aryabhattai* B8W22^T^	EF114313	100.0	Firmicutes	31	53	2
CH-23	*Psychrobacillus* *psychrodurans* DSM11713^T^	AJ277983	99.2	Firmicutes	29	50.51	5
CH-32	*Paenibacillus* *castaneae* Ch-32^T^	EU099594	100.0	Firmicutes	30	52	2
CH-15	*Paenibacillus* *tylopili* MK2^T^	EF206295	99.2	Firmicutes	27	46	9
C-12	*Staphylococcus* *epidermidis* ATCC14990^T^	L37605	99.5	Firmicutes	6	13	3
CH-02	*Acinetobacter* *radioresistens* DSM6976^T^	X81666	99.7	γ-Proteobacteria	22	38	2
C-46	*Lonsdalea* *quercina* LMG26264^T^	JF311441	99.8	γ-Proteobacteria	17	27,28,29,30,31,32	25
C-06	*Erwinia* *typographi* DSM22678^T^	GU166291	97.6	γ-Proteobacteria	3	4	4
C-13	*Luteimonas* *huabeiensis* HB2^T^	JAAN01000045	99.5	γ-Proteobacteria	7	14	7
CH-19	*Pseudomonas* *frederiksbergensis* JAJ28^T^	AJ249382	99.7	γ-Proteobacteria	28	47,48,49	14
C-04	*Pseudomonas* *putida* NBRC14164^T^	Z76667	99.9	γ-Proteobacteria	2	2,3	9
CH-09	*Pseudomonas* *tremae* CFBP3225^T^	AJ492826	100.0	γ-Proteobacteria	26	42,43,44,45	21

**Figure 5.**
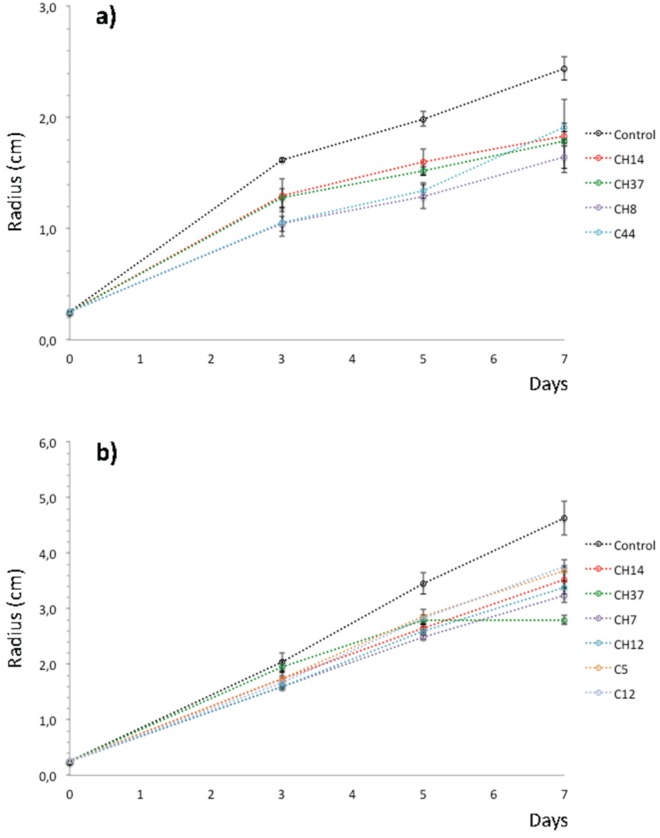
Cumulative radial growth of fungal colonies of *Cryphonectria parasitica* (a) and *Phytophthora cinnamomi* (b) on PDA plates in presence of different bacterial strains from the phyllosphere of chestnut. Data are Mean ± SD (*N* = 3).

Four strains showed consistent antagonism against *C. parasitica*, three of them were isolated from the sampling site CH (CH-8, CH-14 and CH-37) and one from the sampling site C (C-44). After three days of coculture, these four strains significantly (*p* < 0.05) retarded the fungal growth (Figure 5a). After seven days of incubation, these strains reduced the fungal growth by approximately between 22% (C-44) and 33% (CH-8) in relation to growth in control plates. On the other hand, six strains exhibited noticeable antagonistic activity towards *P. cinnamomi*. Two of them were isolated from the sampling site C (C-5 and C-12) and four from the sampling site CH (CH-7, CH-12, CH-14 and CH-37).

With the exception of the strains C-5, CH-14 and CH-37, the other three strains significantly retarded the fungal growth at the third day and thereafter (*p* < 0.05) (Figure 5b). However, strain CH-37 was able to completely stop the growth of *P. cinnamomi* after five days of coculture. At the end of the experimental period, the reduction of fungal growth by these isolates ranged from 29% (C-37) to 15% (C-12). It is noteworthy to point out that strains CH-14 and CH-37 showed antagonism against both *C. parasitica* and *P. cinnamomi*.

We also tested the indole-3-acetic acid (IAA) production ability of the phyllosphere isolates. It was seen that only a small fraction of the bacterial strains were able to produce IAA under the *in vitro* conditions used (data not shown). Noteworthy, none of the strains with antifungal activity exhibited IAA production.

## 4. Conclusions

From chestnut trees growing at two different stands in the mountain range of “Sierra de Tamames-Las Quilamas” (Salamanca province, Spain), we obtained a total of 227 phyllospheric bacterial isolates that grew consistently after cryopreservation and their genetic variability was investigated using random amplified polymorphic DNA techniques. TP-RAPD obtains fingerprints of bacteria using in the PCR two universal primers targeting bacterial 16S rRNA genes at an annealing temperature of 50 °C. TP-RAPD fingerprints have an advantage for grouping purposes because strain-dependent variations are minimal. Thus, strains belonging to the same bacterial taxon (phylotype) share a unique band pattern, which, in turn, is different from that of other bacterial taxa [31]. Therefore, this technique is a good tool for grouping bacteria in order to select representative strains for 16S rRNA gene sequencing, as it has been demonstrated with a broad range of eubacteria [46],[47],[48]. The diversity of strains within each TP-RAPD group was then assessed by M13-RAPD fingerprinting. Like other PCR fingerprinting techniques (i.e. ERIC-PCR, Box-PCR, Rep-PCR), M13-RAPD fingerprinting is an effective technique to distinguish closely related bacterial strains [32],[49],[50]. Since M13-RAPD fingerprinting reveals more subtle genetic differences between similar strains, it gives the possibility to define strain-level OTUs while the TP-RAPD fingerprinting technique does so at phylotype level (genus or species). As indicated by Good's coverage values (Table 1) as well as by Chao1 estimations and rarefaction curves (Figure 2), the total bacterial diversity was highly represented in the samples and the most dominant members of the bacterial communities were likely sampled at both strain level (M13-RAPD patterns) and higher hierarchical levels (TP-RAPD patterns).

According to Shannon's and Simpson's diversity indices (Table 2), the diversity at strain level was significantly greater in the C assemblage than in the CH assemblage. This is a consequence of that both the richness and evenness components of the diversity were higher in the C than in the CH assemblage. Thus, not only the assemblage C was richer in bacterial genotypes (44 vs. 34 M13-RAPD patterns), but also the distribution of isolates amongst the different genotypes was more balanced in the assemblage C than in the assemblage CH (Figure 3a, 3b). At phylotype level (TP-RAPD patterns), however, the richness in the two bacterial assemblages was more similar (26 vs. 23 TP-RAPD patterns) but the distribution of isolates was more equitable in the assemblage CH than in the assemblage C (Figure 3c, 3d). Therefore, the phylotype diversity was lower in the C assemblage than in the CH assemblage, although the difference only reached statistical significance if the diversity is calculated by the Simpson's index (Table 2). This discrepancy between indices could be explained by the fact that the Shannon index stresses the richness component and rare OTUs, whilst the Simpson index lays greater emphasis on the evenness component and on the dominant OTUs.

From all the strains and phylotypes, only 47% of the strains and 58% of the phylotypes were found at both sampling sites. This indicates that the variability in the culturable bacterial communities of the phyllosphere of chestnut is great, even between tree populations geographically close to each other, such as those here studied (approximately 1.5 km apart). It is in agreement with previous studies that have shown that the phyllosphere community structure can vary markedly on the same plant species in different places [51],[52]. In contrast, it has been observed minimal geographic differentiation in the bacterial communities on *Pinus ponderosa* needles even across continents [53]. It should be highlighted that the diversity at strain level was higher in the bacterial assemblage C than in the CH assemblage, while the opposite tended to occur at phylotype level. Given the close proximity between the two sampling locations, the most different environmental factor is the amount of solar radiation received at each one. Due to their different orientations, the rate of insolation is higher at the sampling site CH (oriented towards the southeast) than at the site C (oriented towards the north). Ultraviolet radiation is known to alter the phyllosphere bacterial community composition and gene expression [30],[54],[55]. Moreover, the level of resistance to UV can vary between closely related bacterial strains [56],[57]. Thus, UV resistance was found to be strain specific in *Pseudomonas syringae* pv. *syringae*, being conferred by plasmid-borne determinants homologous to *rulAB* genes for UV radiation resistance. When tested under field conditions, *P. syringae* pv. *syringae* strains lacking these genetic determinants showed a lower survival under direct solar radiation [58]. The lower diversity at strain level in the bacterial assemblage CH suggests that the more UV-sensitive genotypes within each phylotype could have been removed. Either way, strain (M13-RAPD patterns) diversity indices from both bacterial assemblages were relatively high (Table 2), sometime comparable to those obtained in culture-independent studies of phyllosphere bacterial communities [59]–[62].

The isolates from the phyllosphere of chestnut were affiliated with the phyla Actinobacteria, Firmicutes and Proteobacteria (Figure 4; Table 3), which are also predominant in the phyllosphere of other plant species [63]–[66]. According to their 16S rRNA gene sequences, most of the isolates unambiguously belong to recognized bacterial species (sequence similarities equal to or greater than 90%), whereas some others may represent novel species (<99%) or even genera (<97%) (Table 3). This is in concordance with previous studies suggesting the existence of bacteria unique to the phyllosphere habitat [53],[67]–[70]. In this sense, the strain CH-32 was proposed as a new member of the genus *Paenibacillus*, named *P. castaneae* [47]. Although our study was focused on heterotrophic culturable bacteria, we do know that culturing techniques recover only a minimum fraction of bacterial diversity in comparison with molecular approaches [71] and, therefore, the actual bacterial composition of the rhizosphere of chestnut can be taxonomically much richer.

Despite generally unfavorable environmental conditions in the phyllosphere due to long- and short-term fluctuations in factors such as ultraviolet radiation, nutrient availability, temperature and moisture content [12],[72], a high diversity of bacteria colonizing this habitat has been found in several plant species [51],[63],[73]. In agreement with these and other previous works, our results indicate that the sweet chestnut phyllosphere can also be inhabited by a diverse range of heterotrophic culturable bacteria. However, some noticeable differences regarding the presence and relative abundance of some bacterial genera can be found with other studies. Thus, the relative frequency of isolates belonging to the genera *Dietzia* (15%) and *Lonsdalea* (11%) (formerly *Brenneria* [74]), in the chestnut phyllosphere is the highest in the literature to the best of our knowledge. On the other hand, no representative of the genus *Curtobacterium* was recovered in this study while they are among the most common in the phyllosphere of potato, tomato, resurrection fern, beech and oak [52],[65],[75]. These particularities of the bacterial community structure and composition of the phyllosphere of chestnut trees are consistent with increasing research demonstrating that different plants select for distinct microbial communities [12],[51],[59],[66].

All the bacterial strains were tested for antagonism against the chestnut blight and ink fungi, *C. parasitica* and *P. cinnamomi*. Four strains inhibited in vitro the growth of *C. parasitica*, while six strains did inhibit the growth of *P. cinnamomi* (Figure 5a, 5b). Only two strains (CH-14 and CH-37) showed antagonism against both *C. parasitica* and *P. cinnamomi.* To the best of our knowledge, few bacteria have been reported to show antifungal activity against *C. parasitica*, among which there are strains from the genera *Acinetobacter* [76], *Serratia* [77], *Pseudomonas* [78], and a strain of the species *Bacillus subtilis* that was isolated as endophyte from the xylem sap of chestnut stems [79]. Bacteria showing antagonism against Phytophthoras are more numerous in the literature [80]–[85], and strains with biocontrol activity against *P. cinnamomi* are known in bacterial genera like *Bacillus*, *Enterobacter*, *Serratia*, *Actinomadura*, *Micromonospora*, *Nostoc* and *Pseudomonas* [78],[86],[87],[88]. According to the similarity of 16S rDNA sequences (Table 3), the above-mentioned eight bacterial strains belong to the species *Pseudomonas tremae* (CH-12, CH-14 and CH-37), *P. frederiksbergensis* (CH-8), *P. putida* (C-5), *Commensalibacter intestinis* (CH-7), *Staphylococcus epidermis* (C-12) and *Dietzia kunjamensis* (C-44). The species *P. frederiksbergensis* and *P. putida* are known to fungal antagonistic strains, some of which have shown to be useful as BCA [89],[90],[91]; however, as far as we know, it is here reported for the first time strains of the species *P. tremae*, *C. intestinis*, *S. epidermis* and *D. kunjamensis* with antifungal activities.

IAA synthesis and secretion is a common feature of bacterial epiphytes that has been associated with enhanced nutrient leakage from plant cells, and this increased nutrient availability may better enable IAA-producing bacteria to colonize the phyllosphere and may contribute to their epiphytic fitness. [72]. We found that only a small proportion of the isolates from the chestnut phyllosphere produces IAA *in vitro*. This should not be considered a surprising result if we keep in mind that IAA biosynthesis in bacteria differs significantly depending on the surrounding environment, as demonstrated in *Erwinia herbicola*, in which the expression of *ipdC*, a plant inducible gene involved in IAA biosynthesis, varies significantly according to the leaf microenvironments [92]. Noteworthy, none of the strains with antifungal activity exhibited IAA production *in vitro*. Also, we found these two features to be strain specific. Thus, among the strains affiliated as *P. tremae* there were IAA producers without antifungal activities (strains CH-13 and CH-36), and also a strain with antifungal activities but without IAA production ability (CH-37).

As conclusion, our results demonstrate that the chestnut phyllosphere harbours a great diversity of culturable bacteria and might be a source of valuable biological agents for the control of the main fungal diseases of this economically important tree species.
